# Research progress of targeted therapy combined with immunotherapy for hepatocellular carcinoma

**DOI:** 10.3389/fonc.2023.1197698

**Published:** 2023-05-25

**Authors:** Shuqi Xie, Mengchao Wang, Chuanxiu Zeng, Yan Ou, Lu Zhao, Dong Wang, Liwei Chen, Fanming Kong, Dan Yi

**Affiliations:** Department of Oncology, The First Affiliated Hospital of Tianjin University of Traditional Chinese Medicine, Tianjin, China

**Keywords:** targeted therapy, immunotherapy, research progress, hepatocellular carcinoma, combination therapy

## Abstract

Hepatocellular carcinoma is a common gastrointestinal malignancy with a high mortality rate and limited treatment options. Molecularly targeted drugs combined with immune checkpoint inhibitors have shown unique advantages over single-agent applications, significantly prolonging patient survival. This paper reviews the research progress of molecular-targeted drugs combined with immune checkpoint inhibitors in the treatment of hepatocellular carcinoma and discusses the effectiveness and safety of the combination of the two drugs to provide a reference for the further application of molecular-targeted drugs combined with immune checkpoint inhibitors in clinical practice.

## 1 Background

In 2020, according to statistics, the number of new cases of primary liver cancer was 906,000 worldwide, accounting for the 6th of all malignant tumors, and the number of new deaths was 830,000, accounting for the 3rd of all malignant tumors (1–3). Hepatocellular carcinoma (HCC) accounts for 75%-85% of primary liver cancers (4). HCC is a serious threat to human health, especially in developing countries in Asia, and the main risk factors associated with HCC are viral (chronic hepatitis B and C), metabolic (diabetes and Non-alcoholic fatty liver disease), toxic (alcohol and aflatoxin) and immune system disorders (5). Due to the asymptomatic nature of HCC in its early stages and the lack of specific biomarkers, most patients with HCC are diagnosed with intermediate to advanced stages (6, 7). Surgical resection, liver transplantation, and some local regional treatments such as hepatic artery chemoembolization and radiofrequency ablation are often used as radical treatments for HCC (8). However, only 30%-40% of HCC patients may receive radical treatment, and the remaining 60%-70% of patients can only receive non-radical treatments, such as transarterial chemoembolization and molecularly targeted drugs (9). Despite the great progress of systemic therapies such as molecularly targeted drugs, hepatocellular carcinoma is prone to drug resistance and recurrence, metastasis, and poor prognosis. In recent years, with the continuous research on immune checkpoint inhibitors, molecularly targeted drugs combined with immune checkpoint inhibitors have shown good effects in the treatment of liver cancer and become a research hotspot.

## 2 Anti-tumor mechanism

HCC is a complex disease caused by the sequential accumulation of multiple genomic and epigenomic alterations in hepatocytes through undergoing Darwinian selection (10). The vast majority of these mutations accumulate at any time and are not involved in carcinogenesis, while only a few are considered to be functional “driver” mutations that alter key signaling pathways, thereby gaining a selective advantage. The most commonly mutated genes are TERT (promoter), TP53, CTNNB1, AXIN1, ARID1A and ARID2 (11). These genes can be classified into 6 major biological pathways including telomere maintenance, Wnt/β-catenin, P53/cell cycle regulation, AKT/mTOR, MAP kinase, epigenetic modifiers and oxidative stress (12). Abnormal epigenetic regulation also plays a critical role in hepatocarcinogenesis by altering gene expression through multiple mechanisms, including DNA methylation, histones, chromatin remodeling, and alterations in the levels of small (microRNAs) and long (lncRNAs) non-coding RNAs. There are interactions between mutation-driver genes and between genetic and epigenetic alterations involved in carcinogenesis. Three major subtypes of HCC: (1) chromosomally unstable mitotic and stem cell-like tumors; (2) CTNNB1 mutant tumors that appear immunosuppressed; and (3) metabolic disease-associated tumors, including those characterized by macrophage infiltration and good prognosis Immunogenic subgroups (13).

HCC is a highly vascularized solid tumor with high microvascular density, and angiogenesis is a key process in the development of HCC (14). Anti-angiogenic drugs include MEK/ERK pathway inhibitors, mTOR pathway inhibitors, histone deacetylase inhibitors, EGF/EGFR pathway inhibitors, and HGF/c-Met pathway inhibitors (15). VEGF is considered to be the main mediator of angiogenesis in primary liver cancer. Molecularly targeted drugs bind VEGF and prevent its interaction with receptors, thereby neutralizing its biological activity. Simultaneous inhibition of the immunosuppressive effects of VEGF and its receptor in tumors includes inhibition of dendritic cell (DC) maturation, promotion of immunosuppressive cell infiltration, and enhancement of immune checkpoint molecule expression (16). The interaction between tumor cells and components of the tumor microenvironment is a key factor in the development of HCC. The tumor microenvironment consists of a complex mixture of multiple types of non-malignant cells, extracellular matrix, and signaling molecules that play a key role in tumor progression and response to therapy by inducing inflammation, angiogenesis, hypoxia, and fibrosis (17). Cytokines such as interleukin-1 and interleukin-6, activated by liver macrophages (Kuffer cells) and inflammatory cells, interact with the extracellular matrix to promote hepatocellular carcinoma fibrosis and carcinogenesis. Since HCC is an inflammation-associated tumor, promoting an immunosuppressive environment is a key step in tumorigenesis.

**Figure 1:**
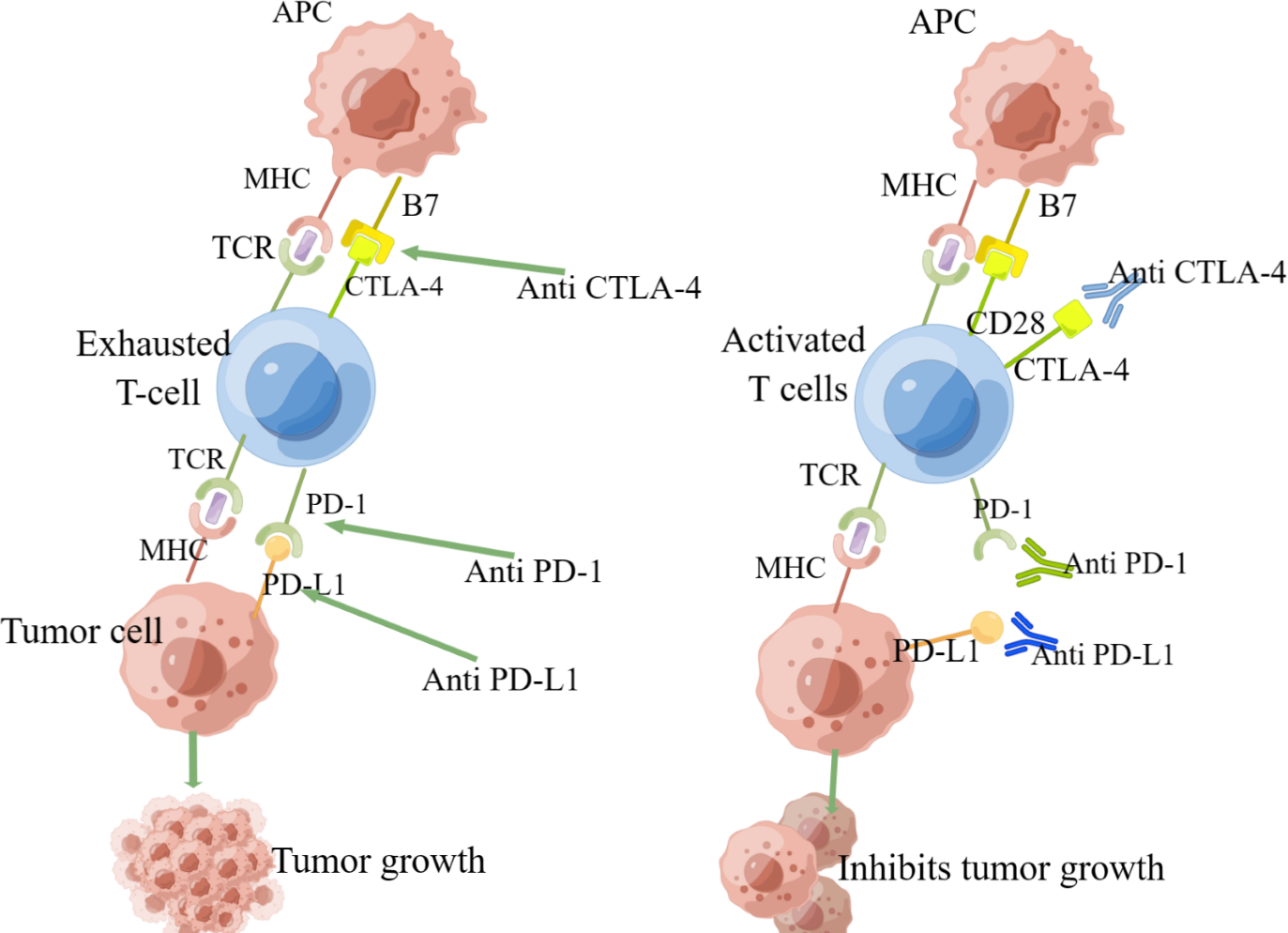
The anti-tumor mechanism of immune checkpoint inhibitors).

Immune checkpoint inhibitors usually act on three targets: (i) cytotoxic T-lymphocyte-associated protein-4 (CTLA-4); (ii) programmed cell death protein receptor 1 (PD-1); and (iii) programmed cell death protein ligand 1 (PD-L1) (18, 19). (Specific drugs are shown in **Table 1**) PD-1 is an immune checkpoint receptor expressed by activated T cells and is an important immunosuppressive molecule.PD-1 binding to ligands leads to the downregulation of T cell receptors and inhibits T cell activation and cytokine release. It regulates the immune system and promotes self-tolerance by downregulating the immune system response to human cells and suppressing the inflammatory activity of T cells (27). PD-1 regulates CD8+ T cell initiation, and anti-PD-1 treatment induces Interleukin(IL)-12 production by intra-tumor DCs, and IL-12 delivery upregulates multiple genes encoding cytotoxic T lymphocyte (CTL) effector molecules (28). PD-L1 is a ligand for PD-1, which is associated with the suppression of the immune system and conducts suppressive signals. The binding of PD-L1 to PD-1 on T cells leads to dephosphorylation of T cell receptors, thereby reducing T cell proliferation and activity (29). Tumor cells can also express PD-L1, and once PD-1 and PD-L1 combine, they will send negative regulatory signals to T cells, resulting in T cells failing to recognize cancer cells and tumor cells thus achieving “immune escape” (30). PD-L1 blockade transiently activates pre-dysfunctional CD8+ T cells by causing co-stimulation of the T cell receptor (TCR) signaling pathway and CD28 at the interface with DCs (31). PD-1 monoclonal antibody or PD-L1 monoclonal antibody blocks the binding of PD-1 to PD-L1, restores T-cell tumor activity, activates T cells, and kills tumor cells (32). CTLA-4 plays a negative regulatory role in the immune system and is mainly expressed in Treg cells. When T cells are activated, CTLA-4 expression is upregulated and the degree of T cell inflammatory response is reduced, thereby enhancing tumor immune escape in hepatocellular carcinoma (33). Drugs that target CTLA-4, PD-1, and PD-L1 can modulate the body’s immune response to exert anti-tumor effects. (As in **Figure 1**).

**Table 1:** Summary of the immune checkpoint inhibitors.

Targets	Drugs			
PD-1	Camrelizumab (20)	Pembrolizumab (21)	Sintilimab (22)	CS1003 (23)
PD-L1	atezolizumab (24)			
PD-1&CTLA-4	Cadonilimab (25)			
PD-L1&CTLA-4	KN046 (26)			

The tumor necrosis factor receptor superfamily (TNFRSF) promotes the activation of immune cells, and clinical trials targeting agonists of OX40 and CD27, members of the TNFRSF family, are underway for the treatment of tumors (34, 35). T-cell immune receptor effectively suppresses innate and adaptive immunity (34). TIM-3 is the most expressed immune checkpoint receptor on tumor NK cells in HCC, and inhibition of TIM-3 enhances the antitumor effect of PD-1 blockers (36). Tryptophan is an essential amino acid that is metabolized primarily through the kynurenine pathway. Indoleamine 2,3-dioxygenase (IDO) and tryptophan 2,3-dioxygenase (TDO or TDO2) are the initiating and key enzymes that catalyze this pathway, and removal of tryptophan inhibits T cell proliferation and activity (37, 38). Thus, small molecule inhibitors of the kynurenine pathway and IDO are expected to be potential tumor immunotherapy agents. Lymphocyte activation gene 3 (LAG-3) is an immunosuppressive receptor and fibrinogen-like protein 1 (FGL1) is a major LAG-3 functional ligand, which is low in normal hepatocytes and significantly elevated in HCC cells (39). Therefore, the FGL1/LAG-3 pathway could be a potential target for immune escape and cancer immunotherapy. In addition to ICI, immunotherapeutic strategies for HCC patients have targeted therapies to promote antibody-dependent cell-mediated cytotoxicity (ADCC), pericyte therapy (ACT), including autologous CD8 T cells, iNKT cells, γδ T cells, cytokine-induced immune killer cells (IKC), chimeric antigen receptor (CAR)-T cells, transfer of lysing viruses and vaccines (40).

Molecularly targeted drugs reduce VEGF-mediated immunosuppression in tumors and their microenvironment and enhance the efficacy of anti-PD-1 and anti-PD-L1 by reversing VEGF-mediated immunosuppression and promoting intra-tumor T-cell infiltration, thereby enhancing the efficacy of immune checkpoint inhibitors (41, 42), Molecularly targeted drugs combined with immune checkpoint inhibitors have additive or synergistic antitumor effects, and therefore targeted combination with immunization may be an effective treatment in unresectable HCC (43, 44).

## 3 Clinical applications

### 3.1 Anti-PD-1 antibody drugs combined with molecularly targeted drugs

#### 3.1.1 Camrelizumab in combination with apatinib regimen

A single-arm, open-label, phase II clinical trial (NCT04297202) showed good efficacy and manageable toxicity of camrelizumab in combination with apatinib in patients with resectable perioperative HCC (20). The results of the study confirmed that preoperative target-free neoadjuvant therapy reduced the postoperative recurrence rate of hepatocellular carcinoma and was well tolerated by patients without serious perioperative adverse events (AE). Target-immunity combination therapy as a neoadjuvant option for resectable HCC can reduce the 1-year postoperative recurrence rate and improve the 1-year survival rate without compromising the safety of surgery (45). In addition, several studies have shown that HBV load does not affect tumor response in HCC patients treated with camrelizumab in combination with apatinib (46). The combination of camrelizumab and apatinib for advanced HCC with portal vein tumor thrombosis has good efficacy and controllable side effects, and is worth promoting in the clinic (47).

A phase Ib extension study (NCT02942329) showed that camrelizumab in combination with apatinib demonstrated safety and efficacy as a second-line or late-stage treatment for intermediate-stage HCC. Their objective response rate (ORR), disease control rate (DCR), and median duration of response (DOR) were 50.0% (95% CI: 24.7% to 75.4%), 93.8% (95% CI: 69.8% to 99.8%) and 3.4 months (range: 1.4-9.7 months) (48, 49).

A non-randomized, open-label phase II RESCUE trial (NCT03463876) showed that camrelizumab in combination with apatinib showed good efficacy and a manageable safety profile in the treatment of both first- and second-line patients with advanced HCC, with median progression-free survival (PFS) for the two cohorts of 5.7 months (95% CI: 5.4% to 7.4%) and 5.5 months (95% CI: 3.7% to 5.6%), respectively. The 12-month survival rates were 74.7% (95% CI: 62.5% to 83.5%) and 68.2% (95% CI: 59.0% to 75.7%), respectively (50). 77.4% of patients reported ≥ grade 3 treatment-related adverse events (TRAE), the most common of which was hypertension (34.2%), but hypertension can be controlled with antihypertensive medications and generally does not lead to hospitalization or life-threatening conditions; 28.9% of patients experienced serious treatment-related adverse events (TRAE), the most common serious TRAE being increased gamma-glutamyltransferase and neutropenia, and 1.1% of patients experienced treatment-related death (51).

A randomized, international, multicenter phase III SHR-1210-III-310 study showed that camrelizumab in combination with apatinib showed better efficacy and safety than sorafenib in patients with unresectable or metastatic HCC who had not received prior systemic therapy. Median overall survival (OS) was 22.1 months (95% CI: 19.1% to 27.2%) and 15.2 months (95% CI: 13.0% to 18.5%) in the camrelizumab combined with apatinib and sorafenib groups, respectively, and PFS was 5.6 months (95% CI: 5.5% to 6.3%) and 3.7 months (95% CI: 2.8% to 3.7%) in the camrelizumab combined with apatinib and sorafenib groups, respectively. This study demonstrated that camrelizumab in combination with apatinib reduced the incidence of risk of disease progression or death (48.0%), resulting in a survival benefit for the global advanced HCC population (52). Different targeting drugs were used in the experimental and control groups, but apatinib is a small molecule targeting drug against VEGFR-2, which blocks downstream signal transduction by highly selective competition for the ATP binding site of VEGFR-2 (also known as FLK-1), inhibits tyrosine kinase production thereby inhibiting neoangiogenesis in tumor tissue, and finally achieves the purpose of tumor treatment (53). And sorafenib can act on tumor cells and tumor blood vessels at the same time. It has dual anti-tumor effects: it can directly inhibit tumor cell proliferation by blocking the cell signaling pathway mediated by RAF/MEK/ERK, and indirectly inhibit tumor cell growth by blocking the formation of tumor neovascularization through inhibiting VEGFR and platelet-derived growth factor (PDGF) receptors (54). Sorafenib has more targets with stronger antitumor effects than apatinib, so it is more likely to demonstrate the efficacy of targeted combination immunotherapy for advanced hepatocellular carcinoma. This difference does not lead to unreliable results. This study is the first international multicenter phase III clinical study of anti-PD-1 monoclonal antibodies in combination with an anti-vascular targeted small molecule tyrosine kinase inhibitor on PFS and OS outcomes over sorafenib, and camrelizumab in combination with apatinib treatment achieved the longest OS benefit to date. In patients with HCC with a large tumor burden that cannot be resected, advanced hepatic artery chemoembolization combined with AC has better survival than early combination (55, 56).

These trials achieved good ORR for camrelizumab in combination with apatinib for HCC, prolonged OS, and presented a reasonable safety profile. In the IMBrave150 trial, the incidence of upper gastrointestinal bleeding observed in the atezolizumab combined with the bevacizumab group was 7%, as bleeding is a known AE with bevacizumab. In contrast, a single-center retrospective study of 38 patients showed that only 3.2% of patients experienced upper gastrointestinal bleeding during “C+A” treatment (57). Although the base of 38 patients is small and limited by the lack of data, it can still serve as a reference value. These trials may be limited by the absence of a control group, a small trial base, and a lack of quality-of-life data.

#### 3.1.2 Pembrolizumab in combination with regorafenib regimen

A phase Ib dose-escalation study (NCT03347292) included 57 patients, 35 patients started oral REG 120 mg/day and 22 patients started oral REG 80 mg/day, plus a fixed dose of PEMBRO 200 mg IV, respectively. Of the 35 patients treated with REG 120 mg, 10 (31%) had a partial response (PR) and 18 (56%) had stable disease (SD); DCR was 88%. Of the 22 patients taking REG 80 mg, 4 (18%) had a PR and 16 (73%) had SD; DCR was 91%. Grade 3/4 treatment-emergency adverse events (TEAE) occurred in 86% of REG 120mg patients and 50% of REG 80mg patients. The most common grade 3/4 TEAE in the REG120 mg/80 mg group were elevated aspartate aminotransferase (AST) (23%/9%), elevated lipase (20%/5%), elevated glutamate aminotransferase (ALT) (17%/9%), and elevated hypertension (17%/9%) (21), the incidence of grade 4 drug-related adverse events was low, no treatment-related bleeding was reported, and AEs that occurred during treatment were manageable.

This trial demonstrated that pembrolizumab in combination with regorafenib had a favorable clinical efficacy and safety profile. The 80 mg/d group had a better safety profile and lower rates of drug-related dose reductions and discontinuations compared to the regorafenib 120 mg/d group. In terms of efficacy, the combination of regorafenib and pembrolizumab showed better antitumor activity than both monotherapies.

#### 3.1.3 Sintilimab in combination with bevacizumab biosimilar regimen

The phase Ib single-center (NCT04072679) study showed that sintilimab in combination with a bevacizumab biosimilar (IBI305) showed good efficacy and better resistance in the treatment of advanced HCC.34% of its patients achieved PR, 44% achieved SD, 22% had disease progression (PD), ORR for all patients was 34.0% (95% CI: 20.0%-48.0%), DCR was 78.0% (95% CI: 66.0%-90.0%), PFS was 10.5 months (95% CI: 8.3% to 12.7%), OS of 20.2 months (95% CI: 16.1% to 24.3%), and DOR of 13.2 months (95% CI: 5.9% to 20.4%). The most common TEAEs were hypertension (32.0%), proteinuria (26.0%), and fever (26.0%). The most common possible immune-related adverse events (IRAE) were fever (26.0%), hypothyroidism (24.0%), myalgia (20.0%), and rash (18.0%) (58). Studies have shown that both serum CD137 concentrations and tumor infiltration by M1 macrophages can be used as potential predictive biomarkers, with both high serum CD137 concentrations and high-density M1 macrophage infiltration in the tumor stroma significantly associated with better outcomes, prolonged PFS and OS (59).

The phase II ORIENT-32 study (NCT03794440) showed that sintilimab in combination with a bevacizumab biosimilar (IBI305) showed good efficacy and a reliable safety profile in patients with unresectable HCC with no prior systemic therapy. Patients receiving intravenous sintilimab (200 mg every 3 weeks) and intravenous IBI305 (15 mg/kg every 3 weeks) had an ORR of 25.0% (95% CI: 9.8% to 46.7%) and a 29% rate of grade 3 or worse TRAE, with a safety profile similar to that of atezolizumab in combination with bevacizumab (22).

The phase III ORIENT-32 study (NCT03794440) showed significant OS and PFS benefits of sintilimab in combination with a bevacizumab biosimilar (IBI305) versus sorafenib in the first-line treatment of patients with unresectable HBV-associated HCC. The median PFS for patients in the sintilimab-bevacizumab biosimilar group was 4.6 months (95% CI: 4.1% to 5.7%) significantly longer than the 2.8 months in the sorafenib group (HR = 0.56, 95% CI: 0.46% to 0.70%; p < 0.0001). Sintilimab-bevacizumab biosimilar had significantly longer OS than sorafenib (median not reached [95% CI: not reached-not reached)] vs 10.4 months [8.5 -not reached]; HR=0.57,95% CI: 0.43% ~0.75%; p < 0.0001). The time to disease progression was significantly longer in patients using sintilimab combined with bevacizumab biosimilar (6.7 months, 95% CI:5.5% to 7.3%) than in the sorafenib group at 4.1 months (95% CI:2.9% ~5.2%; HR= 0.73, 95% CI:0.56% 0.94%) (60). Sintilimab combined with bevacizumab biosimilar treatment significantly reduced the risk of death and disease progression. The most common grade 3-4 TEAEs are hypertension and palmar-plantar red sensory disorder syndrome with manageable treatment-related adverse effects (22).

The above trials illustrate that sintilimab combined with bevacizumab biosimilar is significantly better than sorafenib in terms of efficacy and significant anti-tumor effect. The incidence of grade 3-4 TRAEs in the combination group was comparable to that in the sorafenib group in terms of safety, at 33.7% and 35.7%, respectively, but considering that the duration of dosing in the combination group was approximately twice that in the sorafenib group (median duration of treatment: 6.6-7.0 months vs. 3.5 months), the incidence of grade 3-4 adverse events per unit of time in the combination group was only half that of the sorafenib group. AEs that did not affect the quality of life (hypertension, proteinuria, and platelet reduction) occurred more frequently in the combination group (61). Therefore, the combination therapy group had less impact on patient’s quality of life compared to the sorafenib group.

#### 3.1.4 CS1003 in combination with lenvatinib regimen

A phase Ib (NCT03809767) study showed that CS1003 in combination with LEN showed good antitumor activity and an acceptable safety profile in Chinese patients with first-line unresectable HCC. Patients received CS1003 200 mg IV once every 3 weeks and LEN orally (weight ≥ 60 kg: 12 mg/day; < 60 kg: 8 mg/day). Of 20 patients evaluated for efficacy, ORR was 45.0%, and 9 patients achieved PR. As of the data cut-off date (4.2 to 18.7+ months), the median DOR was not achieved. The DCR was 90.0%, with the best SD in 9 patients. Median PFS was 10.4 months (95% CI: 6.2 to not estimable). 6- and 12-month PFS rates were 85.0% and 48.2%, respectively. Median OS has not been reached. All AEs were grades 1-3. Grade 3 AEs occurred in 9 (45.0%) patients, the most common being elevated gamma-glutamyl transferase (2/20, 10.0%). Grade 3 CS1003-related AEs occurred in 6 patients and grade 3 AEs related to lenvatinib occurred in 4 patients. 2 patients discontinued treatment due to AEs. No patients died due to AEs (23). This trial illustrates that CS1003 has shown good safety and efficacy in the advanced HCC patient population, providing a new option for patients. In addition, CS1003, a novel PD-1 with a unique mechanism, was confirmed to have an ORR of 45% in combination with lenvatinib, which has a very great clinical advantage for advanced non-resectable HCC. More clinical trials will continue to explore the potential of CS1003 in oncology treatment in the future.

In addition, a multicenter, double-blind, randomized, controlled phase III clinical study comparing the efficacy and safety of CS1003 in combination with lenvatinib versus placebo in combination with lenvatinib as first-line treatment for subjects with advanced HCC is underway(CTR20192524).

#### 3.1.5 Pembrolizumab in combination with lenvatinib regimen

A phase Ib multicenter open-label study showed good antitumor activity of pembrolizumab in combination with lenvatinib in advanced HCC, and toxicity was manageable. A total of 104 patients were enrolled, and patients received pembrolizumab 200 mg intravenously once every 3 weeks and lenvatinib orally (weight ≥ 60 kg: 12 mg/day; < 60 kg: 8 mg/day). The median follow-up was 10.6 months (95% CI, 9.2 ~ 11.5 months). The ORR was 46.0% (95% CI, 36.0%-56.3%), the DORs were 8.6 months (95% CI, 6.9 months to not estimable [NE]), the median PFS was 9.3 months, and the median OS was 22 months. At the time of data cut-off, 37% of pith lenvatinib were collected over two and a half years from February 27, 2017, to October 31, 2019, while the 1-year survival rate for advanced HCC was still less than 50% (62). Secondly, OS and PFS were longer with pembrolizumab in combination with lenvatinib compared to other combination regimens. The most commonTRAEs at all levels were hypertension (36%), diarrhea (35%), fatigue (30%), decreased appetite (28%), and hypothyroidism (25%), with 67% of patients experiencing a grade 3 TRAE (63).

A global, double-blind, placebo-controlled phase III LEAP-002 study to confirm the superiority of lenvatinib in combination with pembrolizumab over lenvatinib alone for the treatment of first-line advanced HCC. Seven hundred and ninety-four patients with systemically untreated advanced hepatocellular carcinoma were enrolled and treated with either lenvatinib + pembrolizumab (“cola combination”) or lenvatinib + placebo (lenvatinib monotherapy) in a 1:1 ratio. The dual primary endpoints were OS and PFS. The protocol specified 2 interim analyses (IAs) and a final analysis (FA) for OS. Prespecified efficacy boundaries were one-sided P = 0.002 for PFS at IA1(prespecified final PFS analysis) and 0.0185 for OS at FA. The study showed that the median follow-up time for OS was 32.1 months (range 28.8-41.1) and the OS rate at 24 months was 43.7% vs. 40.0% in the lenvatinib + pembrolizumab group vs. the lenvatinib monotherapy group, respectively (HR,0.840; 95% CI,0.708-0.997; P=O.0227). The median OS for pembrolizumab + lenvatinib vs lenvatinib was 21.1 and 19.0 months, respectively (HR=0.84, 95% CI 0.708-0.997, P=0.0227). A safety threshold of 0.0185 was not achieved. The median follow-up for PFS was 17.6 months (range 11.3-26.6). 12-month PFS rates were 34.1% and 29.3%, respectively, and 24-month PFS rates were 16.7% and 9.3%, respectively (HR,0.834;95% CI,0.712-0.978). Median PFS for pembrolizumab + lenvatinib vs lenvatinib was 8.2 months and 8.0 months, respectively (HR=0.867, 95% CI 0.734-1.024, P=0.0466) (64). The results were not statistically significant. Despite the wide acceptance of lenvatinib in combination with PD-1 antibody in clinical use, because this study was negative, the ‘cola combination’ will not be approved for first-line treatment of advanced HCC and will not be recommended at a high level of guidelines. However, lenvatinib in combination with PD-1 antibody remains a worthwhile first-line treatment option based on accessibility and high antitumor activity.

A multicenter, double-blind, randomized phase III LEAP-012 study is underway, which evaluates the efficacy of systemic combination TACE in combination with lenvatinib + pembrolizumab compared to TACE alone in patients with intermediate-stage HCC (65). There have been studies showing that neoadjuvant PD-1 targeted immunotherapy coupled with tyrosine kinase inhibitors (TKI) has shown promising efficacy and tolerable mortality in liver transplant recipients under close clinical monitoring (66).

### 3.2 Anti-PD-L1 antibody drugs combined with molecular targeting drugs

#### 3.2.1 Atezolizumab in combination with bevacizumab regimen

Phase 1b GO30140 study (NCT02715531) showed longer PFS with atezolizumab in combination with bevacizumab than with atezolizumab alone in unresectable hepatocellular carcinoma with no prior systemic therapy (24).

The phase III IMBrave150 trial (NCT03434379) showed that in patients with unresectable HCC who had not received prior systemic therapy, 12-month OS was 67.2% (95% CI,61.3-73.1) and median PFS was 6.8 months (95% CI,5.7-8.3) for atezolizumab in combination with bevacizumab, and sorafenib 12-month OS and median PFS of 54.6% (95% CI,45.2-64.0) and 4.3 months (95% CI,4.0-5.6), respectively (67).

The trial results illustrate that atezolizumab in combination with bevacizumab has superior OS and PFS to sorafenib, and the clinical subgroup analysis is consistent with this, and the trial provides strong evidence for the previous phase 1b study. In addition, the median time to the quality of life and liver function deterioration was significantly longer than the median PFS in the atezolizumab combined with the bevacizumab group, a difference not observed in the sorafenib group (67).

A randomized, multicenter, open-label, phase III IMBrave 050 study (NCT04102098) showed that patients in the atezolizumab combined with bevacizumab group had a significantly lower risk of recurrence or death by 28% compared with patients in the active surveillance group. The median follow-up time was 17.4 months, and the HR for relapse-free survival (RFS) was 0.72 (95% CI, 0.56,0.93; P=0.012). IMBrave 050 became the first phase III study to demonstrate the effectiveness of adjuvant therapy in patients with high-risk recurrent HCC who underwent surgical resection or ablation (68). One study showed that atezolizumab plus bevacizumab in combination with hepatic arterial infusion chemotherapy (HAIC-FOLFOX) had significant therapeutic efficacy and manageable AE in patients with advanced HCC, which may be a potential treatment option for advanced HCC (69). Atezolizumab in combination with bevacizumab showed good efficacy and safety in both non-virally infected HCC patients and virally infected patients. There were no significant differences in ORR, DCR, PFS, and AE between the two (70). The combination of atezolizumab with bevacizumab and intensity-modulated radiation therapy (IMRT) for the treatment of HCC is a promising option (71).

#### 3.2.2 Atezolizumab in combination with cobimetinib regimen

A Phase I/Ib, global, multicenter, open study to evaluate the safety and activity of atezolizumab in combination with cobimetinib in patients with solid tumors (NCT01988896). The study consisted of a phase I dose-escalation stage and a phase Ib indication-specific expansion stage. The median duration of their safety follow-up was 4.2 (0.7-40.2) months. The most common AEs were diarrhea (67%), rash (48%), and fatigue (40%), similar to single-agent cobimetinib and atezolizumab. Some durable responses were observed in patients receiving atezolizumab in combination with cobimetinib. Due to the small sample size, it was not possible to determine the safety and activity in unresectable HCC without prior systemic therapy (72).

An open, multicenter, multiple short-term phase II COTEST study evaluated the efficacy and safety of cobimetinib in combination with atezolizumab in advanced solid tumors. Cohort 7 (patients with solid non-melanoma or non-hematologic tumors with prior primary or secondary resistance to anti-PD-1 or anti-PD-L1 agents) is not open for recruitment and therefore the safety and activity cannot be determined for unresectable hepatocellular carcinoma without prior systemic therapy (73).

#### 3.2.3 Atezolizumab in combination with cabozantinib regimen

The Phase Ib COSMIC-021 study was designed to evaluate the efficacy of cabozantinib in combination with atezolizumab in a variety of solid tumors. The study results showed that the median DOR for advanced HCC was 22.1 months and the DCR was 83%. The median PFS was 5.7 months and the median OS was 19 months. For safety, the incidence of grade 3/4 TRAE was 40% (74).

A multicenter, open-label, randomized phase III COSMIC-312 study suggests that cabozantinib in combination with atezolizumab may be a treatment option for patients with advanced hepatocellular carcinoma, but more research is needed. The median PFS was 6.8 months in their combination treatment group and 4.2 months in the sorafenib group (HR=0.63, 96% CI 0.44-0.91; p=0.0012). Median OS was 15.4 months in the combination therapy group and 15.5 months in the sorafenib group (HR=0.90, 96% CI 0.69-1.18; p=0.44). The most common grade 3 or 4 adverse events were elevated alanine aminotransferase (ALT), hypertension, elevated aspartate aminotransferase (AST), and painful palmoplantar redness and swelling (75).

### 3.3 Dual antibodies in combination with molecularly targeted drugs

#### 3.3.1 Anti-PD-1 & CTLA-4 cadonilimab in combination with lenvatinib regimen

A single-arm, multicenter phase II (NCT04444167) study showed that AK104 plus lenvatinib has shown promising antitumor activity and an acceptable safety profile as first-line therapy for advanced HCC. Patients with advanced HCC with no prior systemic therapy were treated with AK104 (6 mg/kg IV q2w or 15 mg/kg IV q3w) and lenvatinib [8 mg (weight < 60 kg) or 12 mg (weight ≥ 60 kg) PO QD)]. In 18 patients, ORR was 44.4% (8/18), DCR was 77.8%, and median PFS was not yet reached. TRAE occurred in 83.3% of patients (G3 occurred in 26.7% [8/30] and no G4 or TRAEs were leading to death). The most common TRAEs (≥15%) were elevated AST (36.7%), elevated ALT (36.7%), decreased platelet count (33.3%), decreased neutrophil count (30.0%), and elevated bilirubin (26.7%), the majority of which were grade 1 or 2, with manageable TRAEs (25).

#### 3.3.2 Anti-PD-L1 & CTLA-4 KN046 in combination with lenvatinib regimen

An open, single-arm, multicenter phase II KN046-IST-05 (NCT04542837) study showed that KNO46 in combination with lenvatinib demonstrated good antitumor activity and an acceptable safety profile in the first-line treatment of HCC. Patients with unresectable locally advanced or metastatic HCC who had not received prior first-line systemic therapy were treated with KN046 (5 mg/kg Q3W) in combination with lenvatinib (12 mg/day, body weight ≥ 60 kg; or 8 mg/day, body weight < 60 kg) in a 21-day treatment cycle until disease progression, development of intolerance, or 2 years of treatment. The ORR was 57% (95% CI: 34.0% to 78.2%) and DCR was 95% (95% CI: 76.2% to 99.9%). The incidence of adverse events occurring during treatment was 64% and ≥ grade 3 was 20%; the incidence of AEs associated with KN046 treatment was 60% and ≥ grade 3 was 8%. The grade ≥3 TRAEs associated with KN046 treatment were interstitial pneumonia (n=1,4.0%) and decreased platelet count (n=1,4.0%) (26).

Summary of clinical trials of targeted therapies combined with immunotherapies in **Table 2**.

**Table 2:** Summary of clinical trials of targeted therapies combined with immunotherapies.

Study	Phase	Protocol	ORR	DCR	OS	PFS
			EA/CA	EA/CA	EA/CA	EA/CA
	Ib	Camrelizumab +Apatinib	50%	93.8%		
RESCUE	II	Camrelizumab +Apatinib		74.7%		5.7
SHR-1210-III-310	III	Camrelizumab +Apatinib			21.1/15.2	5.6/3.7
	Ib	Pembrolizumab +Regorafenib		88%		
	Ib	Sintilimab +IBI305	34.0%	78.0%	20.2	10.5
	II	Sintilimab +IBI305	25.0%			
	III	Sintilimab +IBI305				4.6/2.8
	Ib	CS1003 +Lenvatinib	45.0%	90.0%		10.4
	Ib	Pembrolizumab +Lenvatinib	46.0%		22	9.3
LEAP-002	III	Pembrolizumab +Lenvatinib			32.1	17.6
IMbrave150	III	Atezolizumab +Bevacizumab				6.8/4.3
	II	AK104 +Lenvatinib	44.4%	77.8%		Not reached
KN046-IST-05	II	KN046 +Lenvatinib	57%	95%		
COSMIC-021	Ib	Atezolizumab +Cabozantinib		83%	19	5.7
COSMIC-312	III	Atezolizumab +Cabozantinib			15.4/15.5	6.8/4.2

## 4 Prospect

In recent years, molecular targeted therapy combined with immune checkpoint inhibition has made a major breakthrough in the treatment of HCC, and the results of clinical trials have shown sustained clinical benefit with safe and manageable effects. Although survival in HCC has been greatly extended, predicting treatment efficacy and response remains a challenging bottleneck. Due to the heterogeneity of tumor antigens within individual tumors, between tumors in the same patient, and between tumors in different patients, not all tumors respond to immunotherapy in combination with targeted therapies; many tumors that initially respond will eventually become resistant. No validated biomarkers have been identified for tumor response to immunotherapy combined with targeted therapy in HCC patients, nor have the available biomarkers been validated to facilitate clinical decision making. Further research is needed on biomarkers and alternative predictors, including traditional tumor markers, precise checkpoint targets or pathways, tumor mutational burden (TMB), and circulating tumor cells, to accurately identify patients for appropriate treatment. In addition, the use of combination therapies in the treatment of HCC requires further investigation, such as the combination of multiple targeted agents, antitumor agents, and immune or metabolic checkpoint inhibitors with external radiotherapy, hepatic artery chemoembolization, and hepatic artery infusion chemotherapy. The combination of multiple approaches is expected to be effective in killing HCC lesions. With controlled side effects, the quality of life of patients has been greatly improved and patients’ life expectancy has been extended.

## Author contributions

SX and MW conceived of and designed the work. CZ, YO, LZ, DW, LC, DY drafted, and revised the manuscript. All authors contributed to the article and approved the submitted version.
