# Corrigendum: Research progress of targeted therapy combined with immunotherapy for hepatocellular carcinoma

**DOI:** 10.3389/fonc.2023.1338060

**Published:** 2023-12-13

**Authors:** Shuqi Xie, Mengchao Wang, Chuanxiu Zeng, Yan Ou, Lu Zhao, Dong Wang, Liwei Chen, Fanming Kong, Dan Yi

**Affiliations:** Department of Oncology, The First Affiliated Hospital of Tianjin University of Traditional Chinese Medicine, Tianjin, China

**Keywords:** targeted therapy, immunotherapy, research progress, hepatocellular carcinoma, combination therapy

**Text Correction**

In the published article, there was an error in section 3.1.5 Pembrolizumab in combination with lenvatinib regimen

This sentence previously stated: “A multicenter, double-blind, randomized phase III LEAP-012 study showed no significant improvement in the efficacy of systemic combination TACE in combination with lenvatinib + pembrolizumab compared to TACE alone in patients with intermediate-stage HCC.”

The corrected sentence appears below:

“A multicenter, double-blind, randomized phase III LEAP-012 study is underway, which evaluates the efficacy of systemic combination TACE in combination with lenvatinib + pembrolizumab compared to TACE alone in patients with intermediate-stage HCC.”

The authors apologize for this error and state that this does not change the scientific conclusions of the article in any way. The original article has been updated.

